# Establishing percentiles for phase angle, skeletal muscle mass index, and muscle-to-fat ratio in Brazilian adolescents: a bioelectrical impedance analysis study with 59,000 participants

**DOI:** 10.3389/fnut.2025.1686037

**Published:** 2025-12-23

**Authors:** Fernando Guimarães Teixeira, Flávio Andrade Amaral Motta, Alberto Souza de Sá Filho, Iransé de Oliveira Silva, Patrícia Sardinha Leonardo, Rodrigo Alvaro Brandão Lopes-Martins

**Affiliations:** 1 Programa de Pós-Graduação em Ciências do Movimento Humano e Reabilitação, Universidade Evangélica de Goiás—UniEvangélica, Anápolis, Brazil; 2 Hospital do Câncer de Muriaé—Fundação Cristiano Varella, Muriaé, Brazil; 3 Faculdade de Medicina, Afya Centro Universitário Itaperuna, Itaperuna, Brazil; 4 Programa de Pós-Graduação em Bioengenharia, Universidade Brasil, São Paulo, Brazil

**Keywords:** adolescents, body composition, BIA, reference values, phase angle, bioelectrical impedance analysis

## Abstract

**Background:**

Reliable normative references for adolescent body composition are essential for screening and surveillance, yet Brazilian data at national scale remain scarce and heterogeneous across methods and devices.

**Objectives:**

To generate age- and sex-specific reference curves for phase angle (PhA), skeletal muscle mass index (SMMI), and muscle-to-fat ratio (MFR) in Brazilian adolescents, and to present device- dependent body fat percentage (%BF) percentiles as secondary, interpretation- cautioned outputs.

**Methods:**

We analyzed a frozen, de-identified extract of routine multi- frequency bioimpedance assessments (10.00–19.99 years) spanning all five Brazilian macro-regions and multiple recruitment contexts (schools, clinics/primary care, private practices, gyms/fitness centers, community programs). The extract is produced by a vendor privacy/QA pipeline compliant with LGPD; upstream cleaning logs are not accessible to the authors. Prespecified plausibility and deduplication rules (anthropometry and impedance thresholds; robust BIVA on R/H and Xc/H) were re- applied and yielded no additional exclusions. Sex-specific age curves were modeled with GAMLSS (LMS) to estimate smoothed percentiles (P3, P10, P25, P50, P75, P90, P97). %BF, derived from a proprietary embedded equation, is reported as secondary.

**Results:**

The analytic cohort comprised 59,000 adolescents from all macro-regions and diverse contexts. Median PhA, SMMI, and MFR increased with age in boys and showed the expected attenuated or plateauing patterns in girls across mid- to late adolescence. Percentile spreads widened with age for muscle-related indices, indicating growing interindividual variability. Device-dependent %BF percentiles exhibited age- and sex- specific trajectories consistent with physiological expectations but should be interpreted with caution pending external validation.

**Conclusions:**

We provide national reference percentiles for PhA, SMMI, and MFR that are immediately useful for clinical and public- health applications and less sensitive to device-specific assumptions. %BF curves are offered as secondary and require independent validation against a criterion method before routine use. Future work should confirm regional representativeness and cross-device portability.

## Introduction

Adolescence is a transitional phase between childhood and adulthood, generally defined by the World Health Organization as ranging from 10 to 19 years of age. It encompasses a series of complex biological and psychosocial processes, including growth acceleration, hormonal surges, skeletal maturation, and marked changes in energy balance and body composition (1). These transformations are not only influenced by age and sex, but also by genetic factors, physical activity, dietary habits, and socioeconomic context. Importantly, they occur in a relatively short time span and often follow nonlinear trajectories, with distinct patterns in males and females (2, 3).

As a result, adolescence represents a critical period for evaluating body composition, a set of parameters that reflect the proportions of fat mass, lean mass, skeletal muscle, and total body water. These components serve as sensitive indicators of health and nutritional status, and are closely associated with cardiometabolic risk, physical performance, and psychological wellbeing (4). In this context, body composition assessment is vital for early identification of nutritional disorders such as obesity, undernutrition, or even juvenile sarcopenia—conditions increasingly prevalent in sedentary or undernourished youth populations (5).

Several methods have been validated to estimate body composition in pediatric and adolescent populations, including anthropometry, dual-energy X-ray absorptiometry (DXA), magnetic resonance imaging (MRI), and bioelectrical impedance analysis (BIA). Among these, BIA has gained prominence due to its non- invasive nature, portability, affordability, and strong correlation with reference standard imaging techniques when properly validated (6, 7). These characteristics make BIA particularly suitable for large-scale screenings, school-based health assessments, and routine clinical practice.

Nevertheless, the accurate interpretation of BIA-derived variables requires reference values tailored to the specific population being studied, taking into account regional, ethnic, dietary, and socioeconomic diversity. In countries like Brazil, characterized by vast geographic, cultural, and biological heterogeneity, the absence of such normative standards represents a significant barrier to the effective clinical application of BIA in adolescents. Most reference values currently used are derived from high-income countries with distinct demographic and environmental contexts, thereby limiting their generalizability (8, 9). Furthermore, the few Brazilian studies available often rely on small or localized samples, such as athletes or schoolchildren from specific cities, thus restricting the external validity of their findings.

The lack of large-scale, representative datasets also impairs the development of public health policies aimed at addressing early-onset obesity, malnutrition, or physical inactivity. Without sex-specific and age-stratified reference percentiles, health professionals risk misinterpreting BIA values, leading to diagnostic inaccuracies and suboptimal interventions. Given the nonlinear nature of growth and development during adolescence, it is crucial to create normative curves that reflect biological maturation milestones (10).

Internationally, several countries have developed population-specific BIA reference values for children and adolescents across different regions (e.g., Europe and Asia), providing useful external benchmarks for comparison (11, 12). In Brazil, however, this remains an unmet need. The development of robust percentile curves and Gaussian distribution models based on Brazilian adolescents would not only fill a critical gap in the literature but also support health promotion, clinical diagnosis, physical education programs, and nutritional surveillance.

Therefore, the main objective of this study was to establish percentile references and distribution curves for key body composition parameters in Brazilian adolescents aged 10–19 years, stratified by sex and by 3-year age intervals. Secondary objectives were to provide a descriptive characterization of body composition by sex and age, and to identify patterns of biological development and growth rate. While these references are derived from a nationally distributed Brazilian dataset and are intended primarily for use in Brazil, we recognize that cultural, dietary, ancestral, and environmental differences across Latin America may influence body composition. Accordingly, this work should be viewed as a methodological and implementation model to be adapted and locally validated in other Latin American contexts, rather than directly applied without calibration.

## Methods

### Study design and ethical considerations

Participants were recruited from a nationally distributed dataset of bioelectrical impedance examinations performed between 2019 and 2024. The database includes assessments conducted in multiple Brazilian regions—North, Northeast, Central-West, Southeast, and South—encompassing both urban and rural settings. This broad geographic coverage reflects the cultural, socioeconomic, and biological diversity of the country and enhances the generalizability of the findings. All participants were adolescents aged 10–19 years who met the eligibility criteria. Stratification was performed *a priori* by sex and by three age intervals (10–12, 13–15, and 16–19 years) to account for expected developmental milestones.

The study protocol was submitted to and approved by the Research Ethics Committee of the Centro Universitário FAMINAS, in accordance with the principles of the Declaration of Helsinki and the Brazilian Resolution 466/12 on research involving human subjects. All data used in the analysis were anonymized and complied with national data protection regulations (LGPD).

The authors declare that some members of the research team are affiliated with institutions or companies related to the development of bioimpedance technology. To minimize potential bias, several measures were adopted: (i) all data were anonymized and obtained from a pre-existing national database with no influence from the authors on participant selection; (ii) data analysis and statistical modeling were performed independently by researchers with no commercial ties; (iii) the interpretation of results was guided strictly by methodological rigor and current scientific evidence; and (iv) all findings are reported transparently, including limitations. These procedures ensured that the scope, analysis, and conclusions of the study were not unduly influenced by any conflict of interest.

### Participants and inclusion criteria

**Figure 1:**
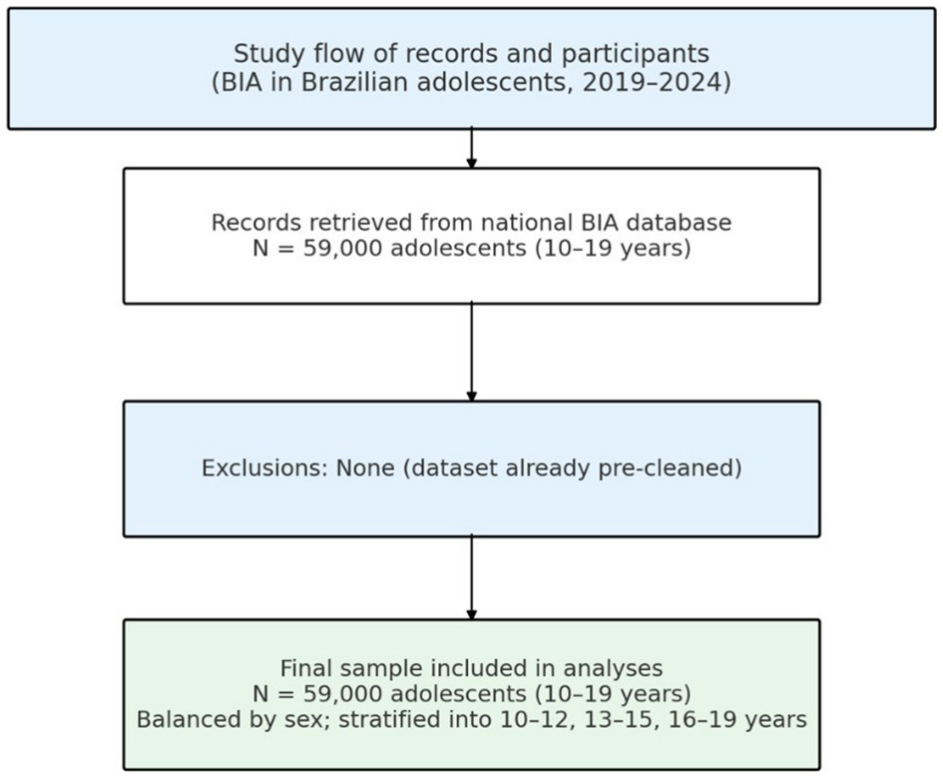
Schematic of data provenance and governance. The operational database of routine assessments undergoes the vendor's standardized privacy/QA pipeline to produce a frozen, de-identified research extract in compliance with LGPD. The prespecified QC thresholds were re-applied by the authors and yielded no additional exclusions; the final analytic cohort included *N* = 59,000 adolescents.

The dataset comprises a total of 59,000 valid BIA examinations of adolescents aged 10 to 19 years (Figure 1).

Recruitment context and national coverage. Records were sourced from a nationally distributed bioimpedance network covering all five Brazilian macro-regions and multiple recruitment contexts (schools, primary care clinics, private practices, gyms/fitness centers, and community programs). Context tags were recorded at the site level and used for descriptive stratification. No recruitment was performed by the authors; rather, we analyzed a frozen, de-identified extract of routine assessments that met the validity criteria defined *a priori*.

Inclusion criteria were: (i) age within the specified range; (ii) complete and technically valid BIA data; (iii) self-declared sex (male or female). Examinations were excluded if they presented implausible values due to measurement artifacts or technical errors, or if participants had known acute or chronic conditions that could affect hydration status or body composition (e.g., severe renal disease, edema, recent hospitalization). For the purpose of analysis, participants were stratified *a priori* by sex and by three age intervals (10–12, 13–15, 16–19 years) to account for expected developmental milestones.

**Figure 2:**
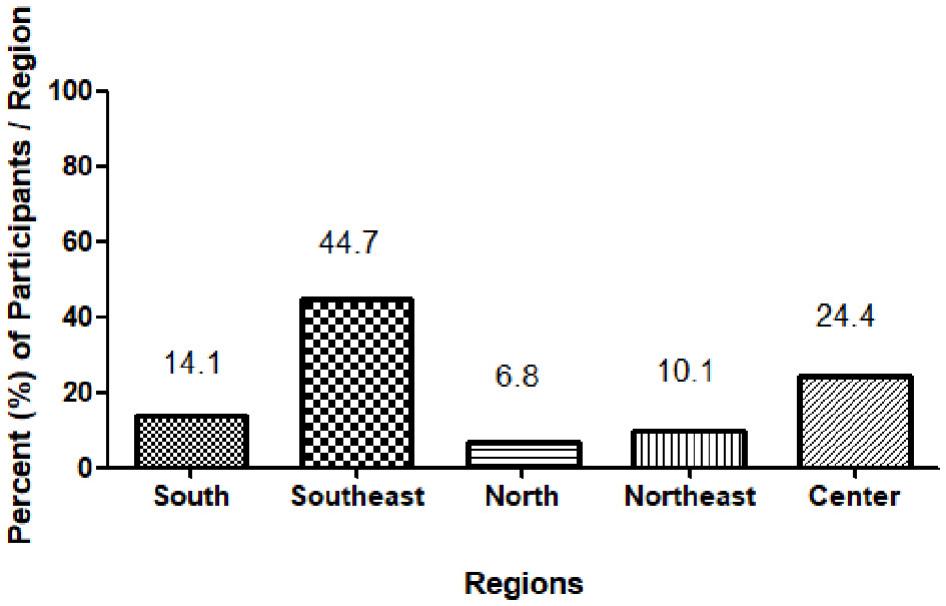
Regional distribution of participants across Brazil. Bars show the proportion of adolescents contributing valid BIA examinations in each macro-region (North, Northeast, Southeast, South, and Center-West; the figure label “Center” refers to Center-West). Numeric labels above each bar indicate exact percentages.

Data-flow overview. Figure 2 depicts data provenance and governance rather than attrition: (i) the operational database of routine BIA assessments undergoes the vendor's privacy/QA pipeline; (ii) a frozen, de-identified research extract is generated; and (iii) our independent QC thresholds are re-applied, yielding no additional exclusions. The final analytic cohort comprises *N* = 59,000 adolescents.

### Data collection and bioimpedance protocol

All BIA assessments were performed using the same multi-frequency segmental analyzer (BIA—Tera Science, São José dos Campos, Brazil). The device operates across a frequency spectrum ranging from 1 to 1,000 kHz, applying safe alternating currents below 500 μA, consistent with international safety standards.

To ensure measurement reliability, the equipment was calibrated routinely according to manufacturer guidelines, with calibration checks performed every 12 months or after ~5,000 tests, whichever occurred first. Acceptable calibration deviations were defined as ≤ 1% for impedance values.

Quality control procedures included: (i) systematic exclusion of repeated exams from the same individual; (ii) removal of measurements with implausible values; and (iii) verification of intra-device stability. Previous pilot data with the same device demonstrated a coefficient of variation (CV) below 2% and a technical error of measurement (TEM) < 1.5% for repeated measures under standardized conditions, confirming the reproducibility of the method.

The BIA device used provides estimations of total body water (TBW), fat mass (FM), fat-free mass (FFM), skeletal muscle mass (SMM), body fat percentage (BFP), and phase angle (PhA), among other variables. The consistency of the measurements was ensured by excluding repeated exams from the same individual and controlling quality standards in data acquisition.

### Quality control and data cleaning

To ensure transparency and reproducibility, we prespecified plausibility and deduplication rules and re-executed them on the frozen analytic extract. A- priori thresholds were: age 10.00–19.99 years; BMI-for-age z-score −5 to +5; height-for-age z-score −6 to +6; standing height 120–210 cm; weight 25–160 kg; phase angle (PhA) 1.0–12.0 °; device-reported %BF 2%−70%. Implausible impedance vectors were flagged using a robust BIVA rule (Minimum Covariance Determinant) applied to R/H and Xc/H. Duplicate assessments were checked via deterministic matching on [site ID, date-time, sex, age (months), height, and weight]. When this pipeline was re-run on the frozen extract, additional exclusions were 0; all records already satisfied the prespecified validity criteria. As the vendor's privacy/QA pipeline produces the de-identified research extract (in compliance with LGPD) and we do not have access to the operational pre- cleaning logs, original upstream exclusion counts cannot be reported.

### Predictive model for body fat percentage

Body fat percentage (%BF) was derived from fat mass (FM) estimated by the device's manufacturer-validated regression model based on bioelectrical impedance variables and anthropometrics. In brief, fat-free mass (FFM) is predicted from height, weight, age, sex, and impedance measurements (resistance/reactance) at multiple frequencies; FM is then obtained as body weight – FFM, and %BF as 100 × FM/body weight. The specific regression coefficients are proprietary to the manufacturer, but the model follows the standard structure used in multi-frequency BIA devices (e.g., FFM = a0 + a1·H^2^/R50 + a2·weight + a3·height + a4·age + a5·sex + ε; where *H* is height and R50 is resistance at 50 kHz), with segmental integration across frequencies. For transparency and reproducibility, we explicitly acknowledge the device-specific nature of the predictive equation and address this as a study limitation in the Discussion.

### Variables and body composition parameters

Device algorithm and transparency. Percentage body fat (%BF) was computed by the multi-frequency BIA device's embedded regression equation. While the general model structure (inputs and computation pathway) is disclosed, regression coefficients are proprietary under a confidentiality agreement with the manufacturer. This is common in commercial analyzers. We therefore treat device-dependent %BF as a secondary outcome and prioritize non-proprietary indices (PhA, SMMI, and MFR) for reference curves and population surveillance.

This study focused on 10 key variables considered essential for the robust characterization of adolescent body composition. These variables are widely recognized in the literature for their clinical relevance and utility in health screening and nutritional assessment. The selected parameters were: age (in completed years); gender (male or female); weight (kg); height (cm); body mass index (BMI, kg/m^2^); body fat percentage (%BF); fat mass (kg); fat-free mass (FFM, kg); skeletal muscle mass (SMM, kg); skeletal muscle mass index (SMMI, kg/m^2^).

Each of these parameters was analyzed separately by sex and by age group (10–12, 13–15, 16–19 years). For all variables, the following statistical descriptors were calculated: mean, standard deviation, and percentiles (P5, P25, P50, P75, P95). Smoothed Gaussian distribution curves were generated to visualize normative variation. The SMMI was adopted to normalize muscle mass values relative to body size and is particularly useful in identifying early sarcopenic tendencies or disproportional body composition.

### Maturation index

A maturation index (MI) was computed as height-to-weight ratio, defined as MI = height (cm)/weight (kg). This proxy was used to capture maturational changes in body size and proportionality over adolescence when Tanner staging was unavailable. Higher MI values reflect taller–leaner profiles typically observed earlier in puberty, whereas lower MI values indicate greater mass relative to stature in later stages.

### Data provenance, governance, and automated QC

Nationwide assessments are streamed daily into a centralized, de- identified pooled ledger managed by the data controller under LGPD-aligned governance. The study team did not have credentials to access the master pool or any site-level logs. A frozen analytic extract was generated by a pre-specified query covering adolescents aged 10–19 years; Figure 1 shows de percentage of participants from the different regions of Brazil.

Data selection and cleaning were fully automated and code-driven, with version-controlled scripts and parameter files. No manual record-level curation was performed by the authors; duplicate resolution followed a deterministic rule (retain the earliest complete record for the same individual). Variables evaluated for QC included sex, age, height, weight, raw resistance (R), raw reactance (Xc), R standardized by height (R/H), Xc standardized by height (Xc/H), device model/firmware, and measurement timestamp.

*A priori* plausibility rules: age outside 10–19.99 y; BMI z < −5 or > +5; height-for-age *z* < −6 or > +6; phase angle < 1 ° or > 12 °; device-reported %BF < 2% or > 70%. Objective multivariate rule: bioelectrical impedance vector analysis (BIVA) using Mahalanobis distance on (R/H, Xc/H) with a robust covariance estimator (minimum covariance determinant); observations with *D*^2^ > $\chi_{0.95,df=2}^{2}$ were flagged as implausible.

### Representativeness checks

Although geographic fields were not available, we assessed heterogeneity using variables present in the extract: sex, 1-year age groups, measurement date (seasonality), device model/firmware, and high-level recruitment context where available (schools, clinics, fitness/gyms, community programs). We summarized body composition indices across these strata to evaluate stability of distributions.

### Statistical analysis

Data were analyzed with computational environment operating system: Ubuntu 22.04 LTS (linux/amd64) Language: Python 3.11.0. LIBRARIES USED: pandas 2.3.1—data manipulation and analysis; numpy 2.3.1—numerical computing; openpyxl 3.1.5—reading/writing excel files; scipy 1.16.0—statistical tests; matplotlib 3.10.3—plots and visualizations; seaborn 0.13.2—advanced statistical visualizations.

Descriptive statistics were calculated for all variables by sex and age group. Normality of distributions was assessed using the Shapiro–Wilk test and visual inspection of histograms. Percentile curves were generated using the Lambda-Mu-Sigma (LMS) method, and smoothed using cubic splines when appropriate. Differences between groups were evaluated using ANOVA or the Kruskal–Wallis test, followed by *post hoc* analysis. A significance level of *p* < 0.05 was adopted.

## Results

### General characteristics and sample distribution

Geographic and context distribution. The analytic cohort included adolescents from all five Brazilian macro-regions and from diverse recruitment contexts (schools, clinics/primary care, private practices, gyms/fitness centers, community programs). Relative participation was highest in the Southeast, followed by the Central-West and South, with smaller contributions from the Northeast and North, reflecting the density of participating centers. The macro- regional and context breakdowns are reported in Table 1. We acknowledge that this asymmetry may influence absolute percentile levels and recommend local calibration where appropriate.

**Table 1:** Sample characterization stratified by sex and age groups.

**Characteristic**	**10–12 years**			**13–15 years**			**16–19 years**		
	**Male**	**Female**	***p*** **-Value**	**Male**	**Female**	***p*** **-Value**	**Male**	**Female**	***p*** **-Value**
Sample size, *n* (%)	2,214 (55)	1,813 (45)	–	7,902 (51.3)	7,487 (48.7)	–	22,023 (55.6)	17,561 (44.4)	–
Age (years)	11.4 ± 0.7	11.4 ± 0.8	892	14.0 ± 0.8	14.0 ± 0.8	156	17.2 ± 1.1	17.1 ± 1.1	< 0.001
**Anthropometric measures**									
Height (cm)	153.7 ± 8.0	154.8 ± 6.7	1	168.6 ± 8.3	161.6 ± 6.1	< 0.001	174.6 ± 6.3	163.1 ± 6.2	< 0.001
Weight (kg)	57.1 ± 14.0	59.1 ± 12.1	< 0.001	65.4 ± 15.3	62.0 ± 13.1	< 0.001	69.2 ± 14.8	62.8 ± 12.9	< 0.001
BMI (kg/m^2^)	24.0 ± 5.0	24.6 ± 4.4	2	23.0 ± 4.7	23.7 ± 4.2	< 0.001	22.6 ± 4.1	23.6 ± 4.0	< 0.001
**Body composition**									
Body fat (%)	18.2 ± 8.9	25.8 ± 7.8	< 0.001	16.1 ± 8.2	24.2 ± 7.5	< 0.001	13.8 ± 7.0	21.8 ± 7.2	< 0.001
Fat mass (kg)	11.8 ± 8.2	16.2 ± 7.1	< 0.001	11.4 ± 8.9	15.8 ± 7.8	< 0.001	10.2 ± 7.8	14.5 ± 7.2	< 0.001
Fat-free mass (kg)	45.3 ± 7.8	42.9 ± 6.2	< 0.001	54.0 ± 8.1	46.2 ± 6.8	< 0.001	59.0 ± 8.5	48.3 ± 6.9	< 0.001
Skeletal muscle mass (kg)	21.8 ± 4.2	19.2 ± 3.1	< 0.001	26.9 ± 4.8	21.8 ± 3.8	< 0.001	31.2 ± 5.2	24.1 ± 4.2	< 0.001
**Body water**									
Total body water (L)	33.2 ± 5.8	31.4 ± 4.6	< 0.001	39.5 ± 6.0	33.8 ± 5.0	< 0.001	43.2 ± 6.2	35.4 ± 5.1	< 0.001
Body water (%)	58.2 ± 6.8	53.4 ± 5.9	< 0.001	60.4 ± 7.1	54.8 ± 6.2	< 0.001	62.8 ± 6.9	56.8 ± 6.1	< 0.001
**Derived indices**									
Muscle/fat ratio	2.2 ± 1.1	1.3 ± 0.6	< 0.001	2.1 ± 1.2	1.0 ± 0.5	< 0.001	2.8 ± 1.5	1.2 ± 0.6	< 0.001
Phase angle (°)	6.2 ± 0.9	5.8 ± 0.8	< 0.001	6.8 ± 1.0	6.1 ± 0.9	< 0.001	7.4 ± 1.1	6.5 ± 1.0	< 0.001
**Metabolic rate**									
Total metabolic rate (kcal)	1,456 ± 198	1,298 ± 156	< 0.001	1,689 ± 218	1,398 ± 178	< 0.001	1,842 ± 245	1,456 ± 189	< 0.001
Percent of participants/region	South 14.1%		Southeast 44.7%		North 6.8%		Northeast 10.1%		Center 24.4%
**Bioimpedance**									
Resistance (Ω)	612 ± 89	658 ± 78	< 0.001	548 ± 82	612 ± 89	< 0.001	498 ± 76	578 ± 85	< 0.001
Reactance (Ω)	67 ± 12	66 ± 11	89	73 ± 14	69 ± 12	< 0.001	81 ± 16	74 ± 13	< 0.001

Data presented as mean ± standard deviation or *n* (%). *p*-values from Mann-Whitney U-test comparing males vs. females within each age group.

A total of 59,000 adolescents aged 10–19 years were included in the final analysis, with balanced representation by sex and across three age categories: early adolescence (10–12 years), mid-adolescence (13–15 years), and late adolescence (16–19 years). All participants had technically valid BIA data and met the inclusion criteria.

Table 1 summarizes the sample characteristics by sex and age group (10–12, 13–15, and 16–19 years), including anthropometry, body composition, and bioimpedance measures. Distributions of %BF, PhA, and MFR were stable across measurement periods and device models. Where context tags were present, summaries were broadly similar between schools, clinics, and fitness settings (Table 1). These findings reduce—though do not eliminate—the risk that the percentiles are driven by a narrow subgroup.

The sample was unevenly distributed across Brazil's macro-regions: nearly half of participants were from the Southeast (44.7%), followed by the Center-West (24.4%), South (14.1%), Northeast (10.1%), and North (6.8%). Percentages sum to 100% with a 0.1-point rounding remainder.

### Age-related trends in body composition by sex

**Figure 3:**
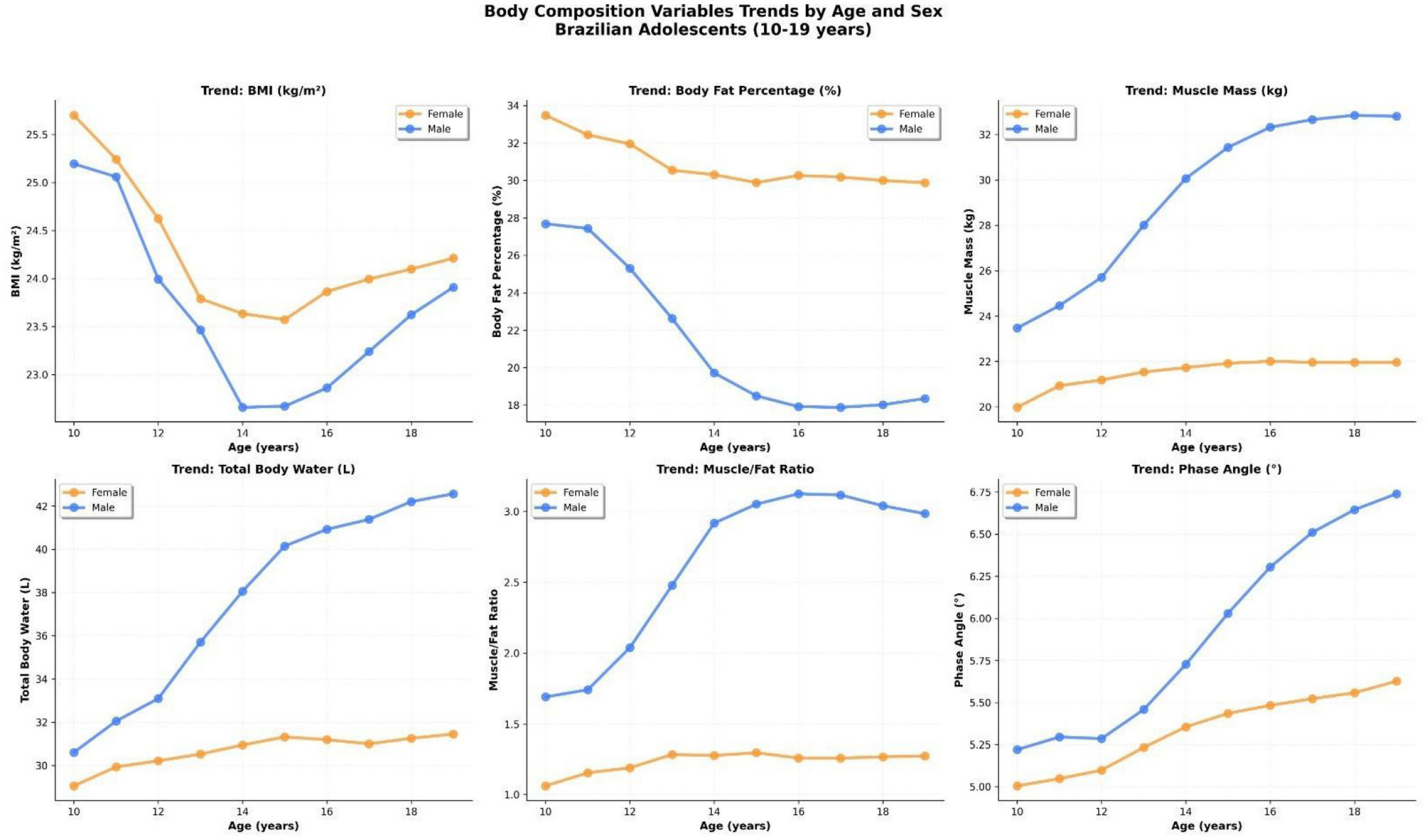
Mean trajectories of six body composition variables by age and sex in Brazilian adolescents (10–19 years). Variables include body mass index (BMI), body fat percentage (%BF), skeletal muscle mass (kg), total body water (L), muscle-to-fat ratio, and phase angle (°). Boys exhibited marked gains in lean mass and phase angle with a concurrent reduction in %BF, while girls maintained more stable patterns.

Figure 3 presents the age-specific mean trajectories of six key body composition variables from 10 to 19 years, stratified by sex. Clear and physiologically consistent divergence was observed between boys and girls during adolescence.

*Body mass index* (BMI) followed a characteristic *pubertal dip* pattern in boys, with a decline between ages 12 and 14 followed by a progressive recovery. This dip likely reflects the adolescent growth spurt, during which height increases rapidly and precedes substantial weight gain. Girls exhibited a more stable BMI trajectory with a mild upward trend from age 14 onward.

*Body fat percentage* (%BF) declined markedly in boys, from ~28% at age 10 to below 20% by age 15, while remaining relatively stable in girls, ranging around 30% throughout adolescence. This sexual dimorphism reflects known hormonal influences, including increased androgen activity in boys leading to muscle hypertrophy and fat redistribution.

*Skeletal muscle mass* increased rapidly in boys, rising from 24 kg at age 10 to over 32 kg at age 18, while girls displayed a more gradual and limited increase, plateauing at around 22 kg. Total body water (TBW) followed a similar trajectory, reinforcing the association between muscle mass and water content, given the high hydration of lean tissue.

*The muscle-to-fat ratio* exhibited a sharp increase in boys, from ~1.5 at age 11 to over 3.0 by age 15, reflecting the disproportionate gain of lean mass relative to fat mass during male pubertal development. In contrast, this ratio remained stable and consistently lower in girls, underscoring sex-based physiological differences.

*Phase angle (PhA)*, a bioimpedance-derived marker of cellular integrity and mass, showed a sustained increase with age in both sexes. However, the magnitude of this rise was more pronounced in boys (from ~5.3 ° to ~6.7 °) than in girls (from ~5.0 ° to ~5.8 °), likely reflecting their higher muscle accretion and cell membrane health.

Together, these trends reveal critical inflection points in adolescent development and underscore the importance of sex-specific reference values for clinical assessment.

### Age-specific normative curves for key anthropometric and body composition parameters

Normative reference curves were developed for BMI, body fat percentage, skeletal muscle mass, and height, stratified by age and sex, using the 25th, 50th (median), and 75th percentiles to reflect central tendency and distribution.

#### Body mass index (BMI)

The BMI curves revealed a downward trend during early adolescence in both sexes, particularly pronounced in boys between 12 and 14 years, a period coinciding with the pubertal growth spurt. Following this dip, BMI gradually increased in both sexes, although the recovery was steeper in males. The interquartile range was broader in boys during mid-adolescence, reflecting greater individual variability in growth and weight gain trajectories.

#### Body fat percentage (%BF)

Interpretation note. The %BF percentiles presented here reflect a device- dependent estimate and should be interpreted with caution until independently validated against a criterion method. For immediate clinical and public health use, we highlight the accompanying reference curves for phase angle (PhA), skeletal muscle mass index (SMMI), and muscle-to-fat ratio (MFR), which are directly derived from raw measurements or transparent transformations. Fat percentage curves exhibited a sharp divergence between sexes beginning at age 12. While girls maintained relatively stable %BF throughout adolescence (median ~30%), boys demonstrated a progressive decline, reaching a plateau near 18%. The female interquartile band remained higher and narrower, suggesting a more homogenous fat distribution in adolescent girls.

#### Skeletal muscle mass

**Figure 4:**
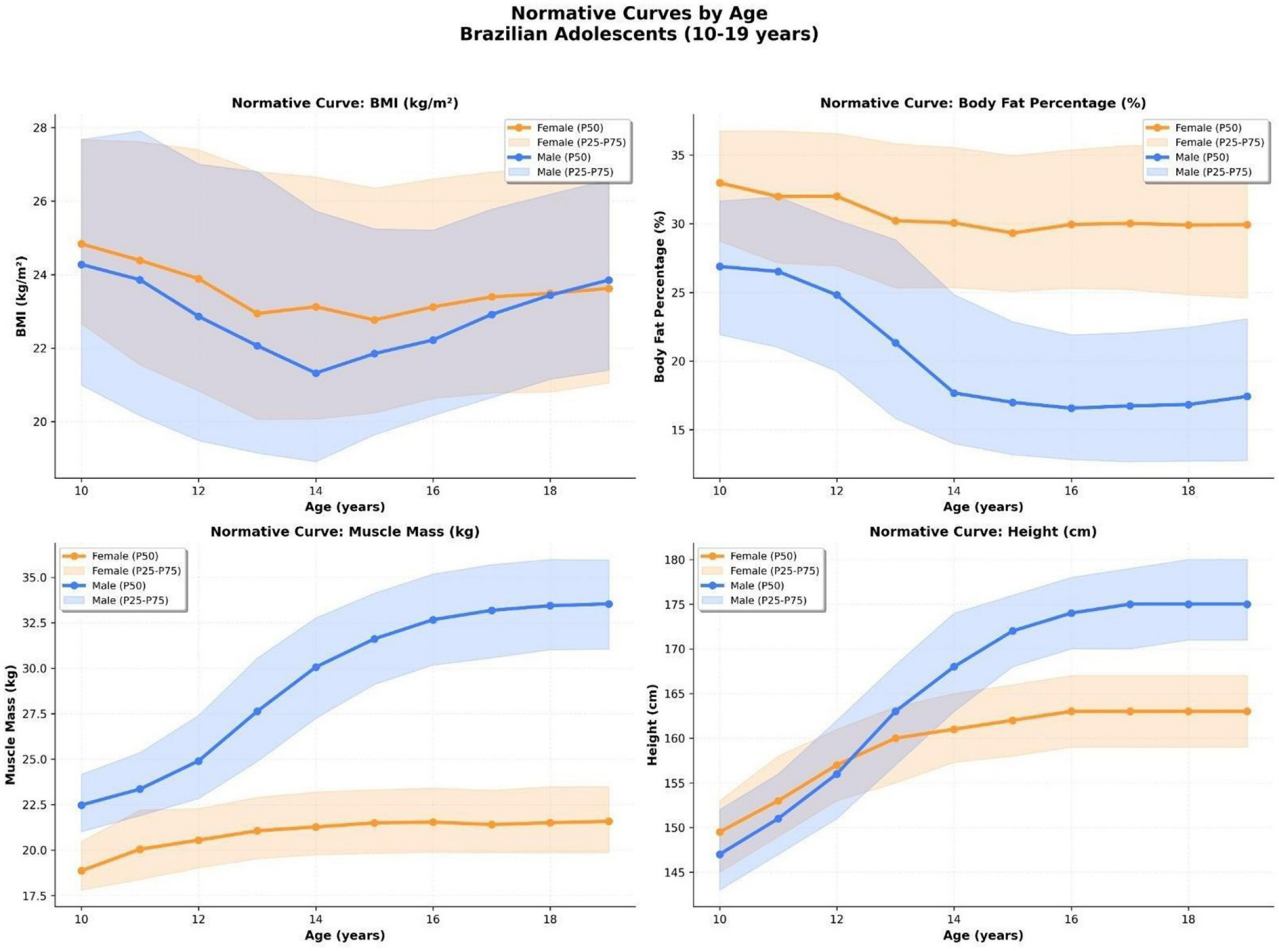
Smoothed percentile curves (P25–P75 and median) for four anthropometric and body composition parameters in Brazilian adolescents aged 10–19 years. Panels show body mass index (BMI), body fat percentage (%BF), skeletal muscle mass (kg), and height (cm), stratified by sex. Curves reflect distinct physiological maturation patterns between boys and girls.

Muscle mass curves showed an accelerated and sustained increase in boys, from a median of ~22 kg at age 10 to over 33 kg by age 18. Girls experienced a slower and more limited increase, reaching a plateau around 22 kg. Notably, the variability among boys widened significantly during puberty, indicating differences in muscle development, timing and intensity (Figure 4).

#### Height

Height curves confirmed classical growth trajectories, with boys surpassing girls around age 13 and continuing to grow at a faster rate until age 17. By age 18, boys reached a median height of ~175 cm, compared to 162 cm in girls. The interquartile spread was also wider in males during the pubertal period, consistent with asynchronous growth patterns.

Together, these normative curves offer a robust reference framework for evaluating body composition and anthropometry in Brazilian adolescents, accounting for the distinct and nonlinear developmental trajectories in males and females (Figure 4).

### Growth velocity and biological development patterns

To better understand the timing and intensity of adolescent maturation, a series of age-specific velocity curves was constructed, capturing changes in height, weight, muscle mass, fat percentage, and the maturation index (height- to-weight ratio). The analyses revealed sex-specific developmental trajectories with clear pubertal inflection points.

#### Height velocity

Height velocity peaked earlier and was more modest in girls (maximum ~5 cm/year at age 11–12), whereas boys reached a higher and delayed peak (~6.5 cm/year at age 13), reflecting classical pubertal timing differences. This aligns with expected hormonal surges in testosterone and growth hormone during mid- adolescence in males (Figure 4).

#### Weight and muscle mass gain

Velocity curves for weight and skeletal muscle mass revealed that boys not only gained more weight, but a larger proportion of it was lean mass. Muscle accretion in boys peaked at age 13 (just above 2.5 kg/year), whereas girls showed lower and more constant gains, stabilizing below 1 kg/year after age 14 (Figure 4).

#### Fat percentage variation

The variation in fat percentage (%BF) presented a divergent trend: boys showed a negative delta between 12 and 15 years (up to −3% per year), suggesting active fat loss relative to total mass during the muscle growth phase. In contrast, girls exhibited a stable or slightly increasing %BF over time, with no significant fat loss phase.

#### Maturation index

The height-to-weight ratio (proxy for biological maturation) was relatively stable in girls but showed a gradual decline in boys after age 14, indicating increased mass gain relative to height in late adolescence, a sign of somatic maturation and metabolic shift toward muscle dominance.

**Figure 5:**
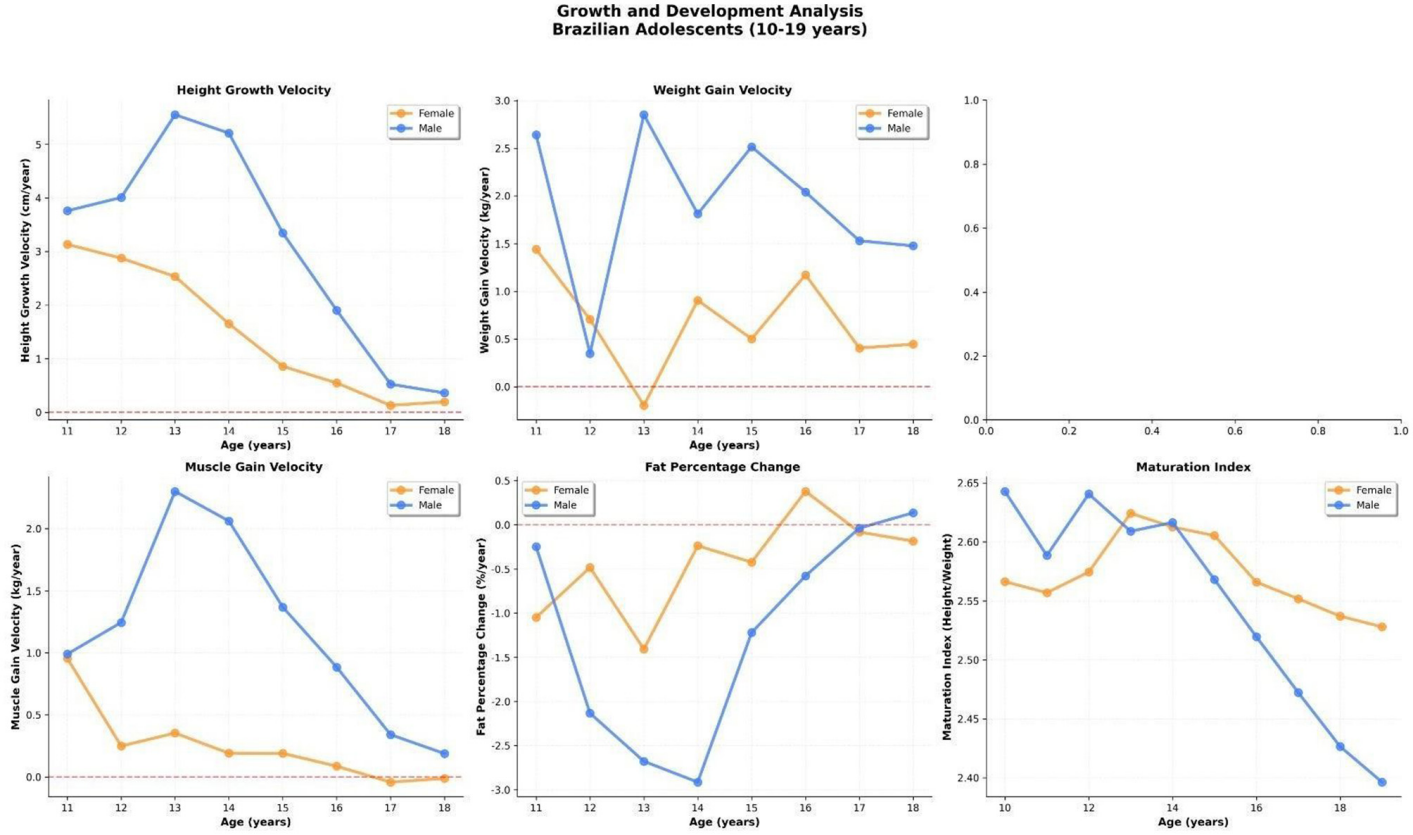
Age-specific velocity curves and biological development indicators in Brazilian adolescents aged 11–18 years. Panels show height velocity (cm/year), weight gain velocity (kg/year), muscle mass gain (kg/year), fat percentage change (%/year), and the maturation index (height/weight ratio), stratified by sex. Boys exhibited delayed but more intense pubertal peaks in height and muscle gain, accompanied by a marked decline in fat percentage.

Together, these findings confirm sex-specific tempo and magnitude of adolescent development and highlight the importance of incorporating growth velocity and dynamic body composition markers into adolescent health assessment frameworks (Figure 5).

### Comparative distributions by sex and age group

**Figure 6:**
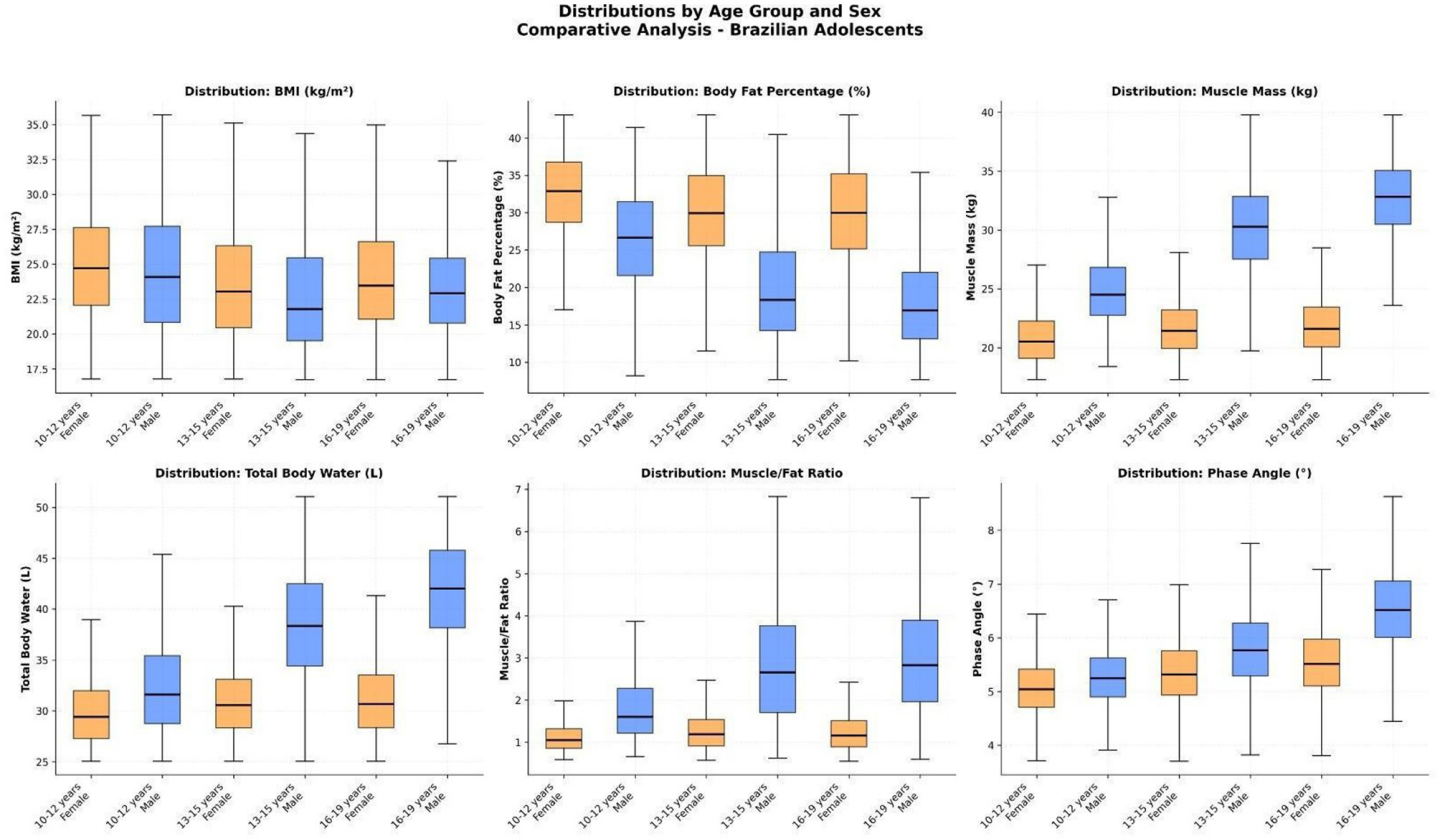
Box-and-whisker plots of eight body composition variables across three age groups (10–12, 13–15, 16–19 years), stratified by sex. Variables include body mass index (BMI), body fat percentage, skeletal muscle mass (kg), total body water (L), muscle-to-fat ratio, and phase angle (°). Plots reveal increasing divergence between boys and girls during puberty, with boys showing greater gains in lean mass and phase angle, and girls maintaining higher %BF.

Boxplots were constructed to compare the distributions of key body composition variables across three age groups (10–12, 13–15, 16–19 years), separately for males and females. This analysis provides a robust visual summary of interindividual variability, central tendency, and sex-based divergence during adolescence (Figure 6).

#### Body mass index (BMI)

BMI distributions were relatively similar between sexes across all age groups. However, slight right-skewness in females was observed in the older group (16–19 years), suggesting greater heterogeneity in body size among older girls.

#### Body fat percentage (%BF)

A pronounced and consistent sex-based gap in %BF was evident in all age categories. Girls had markedly higher median fat percentages than boys, and the interquartile ranges did not overlap, indicating a robust dimorphic pattern. While girls' %BF remained relatively stable across age groups, boys exhibited a downward shift in both median and range over time.

#### Skeletal muscle mass and total body water

For both muscle mass and total body water, boys showed significant increases across age groups, with medians and upper percentiles expanding dramatically from early to late adolescence. In contrast, girls' distributions remained relatively narrow and stable. These variables were especially effective in illustrating the male growth spurt and lean mass accretion.

#### Muscle-to-fat ratio (MFR)

This ratio revealed stark differences between sexes, particularly in the 13–15 and 16–19 age groups. Boys exhibited higher and increasingly skewed MFR distributions, reflecting accelerated muscle gain and fat reduction. Girls displayed lower and more symmetric distributions, with minimal change across age groups.

#### Phase angle (PhA)

Boys demonstrated a consistent upward trend in PhA distributions with age, with both median and range increasing. Girls showed only modest gains, and the difference became more apparent after age 13, mirroring trends in lean mass and cellular integrity.

These findings confirm that compositional divergence between sexes becomes most pronounced during mid-adolescence and remains evident into late adolescence. The widening spread in male distributions also reflects greater biological variability in pubertal timing and muscle growth.

### Kernel density distributions by sex and age group

**Figure 7:**
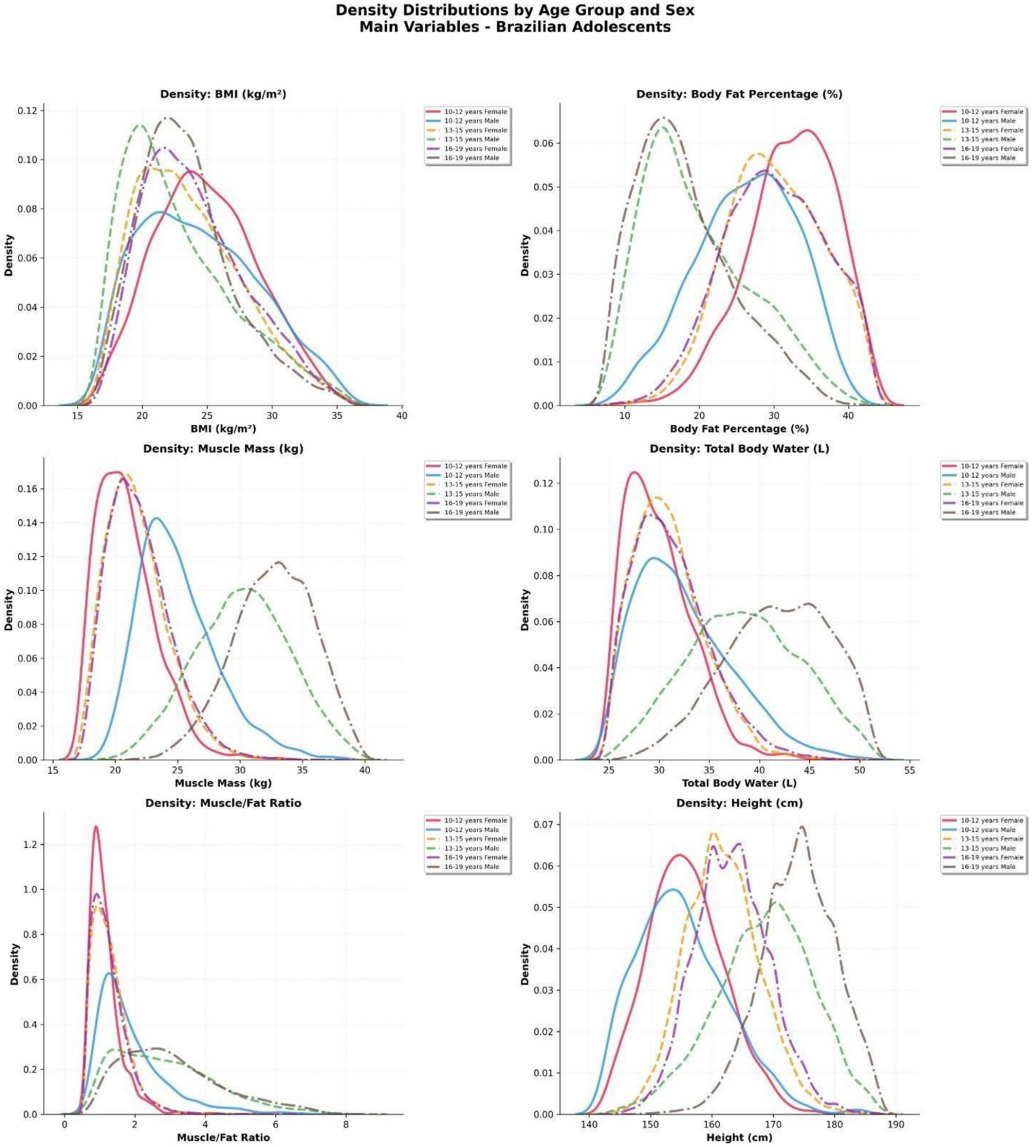
Kernel density curves of body composition and anthropometric parameters in Brazilian adolescents, stratified by sex and age group (10–12, 13–15, 16–19 years). Variables include body mass index (BMI), body fat percentage (%BF), skeletal muscle mass (kg), total body water (L), muscle-to-fat ratio (MFR), and height (cm). Curves illustrate sex-specific developmental shifts and increased population variability during adolescence.

To assess population-level changes beyond central tendency, kernel density estimation (KDE) curves were generated for key body composition and anthropometric variables. These curves illustrate how the entire distribution of values shifts across age groups and between sexes, offering a nuanced view of adolescent development (Figure 7).

#### Body mass index (BMI)

In both sexes, BMI distributions became progressively wider with age. In males, the modal BMI increased gradually from early to late adolescence, reflecting general somatic growth. In females, the modal value remained stable, but right-skewness became more evident in the 16–19 age group, indicating greater heterogeneity and possibly early onset of overweight trends in a subset of the population.

#### Body fat percentage (%BF)

Girls displayed consistently higher and more narrowly clustered %BF distributions in all age groups. In contrast, boys showed a clear leftward shift in %BF distributions with age, from a modal value near 25% in early adolescence to under 20% in the oldest group. This reflects progressive fat loss in males during puberty and the emergence of sexual dimorphism in adiposity.

#### Skeletal muscle mass and total body water

In boys, muscle mass and TBW curves showed rightward shifts and increasing dispersion across age groups, indicating both absolute gains and interindividual variability. Girls' curves remained more compact and stable, with only modest displacement.

#### Muscle-to-fat ratio (MFR)

The MFR density curves in boys were notably right-skewed by mid- and late adolescence, with a minority achieving ratios >4.0, whereas the vast majority of girls remained centered around 1.0–1.5. This visual confirms the growing divergence in lean-to-fat balance between sexes.

#### Height

Height distributions broadened and shifted rightward in both sexes, but more markedly in boys, whose modal height increased from ~155 cm (10–12 years) to ~175 cm (16–19 years). Girls reached their height peak earlier and showed a more stable distribution in later adolescence.

These KDE analyses reinforce the marked redistribution of physiological profiles during adolescence and highlight the emergence of sex-based population phenotypes.

### Relationship between BMI and body fat percentage by sex and age group

A scatterplot analysis was performed to evaluate the association between body mass index (BMI) and body fat percentage (%BF) among Brazilian adolescents, disaggregated by sex and age group.

**Figure 8:**
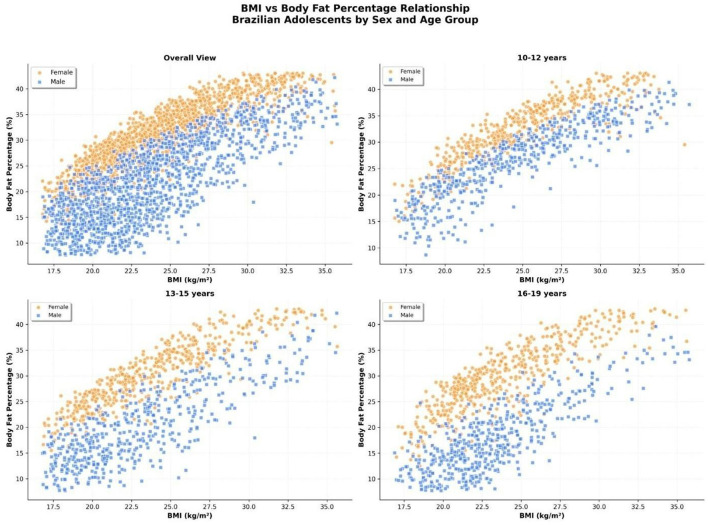
Relationship between body mass index (BMI, kg/m^2^) and body fat percentage (%BF) in Brazilian adolescents (*n* = 59,000), disaggregated by sex (orange = female; blue = male) and age groups (10–12, 13–15, and 16–19 years). Girls consistently exhibited higher %BF than boys at any given BMI, and age- related divergence was observed in the strength and shape of the association.

Overall, a strong positive correlation was observed between BMI and %BF in both sexes (Figure 8). However, at equivalent BMI values, girls consistently presented higher %BF than boys. This sex-related divergence was most prominent in the lower BMI range (18–23 kg/m^2^), where boys exhibited significantly lower fat percentages for a given BMI.

When stratified by age group, the BMI–%BF relationship remained positively linear, but developmental trends emerged:

- 10–12 years: the association was relatively tight for both sexes, with some overlap in the middle BMI range. Girls already demonstrated higher adiposity at every BMI level.
- 13–15 years: the slope of the relationship steepened in boys, with the appearance of leaner profiles at mid-range BMI values. Girls showed a widening of distribution in %BF at higher BMI ranges, indicating increasing body composition heterogeneity.
- 16–19 years: sexual dimorphism became accentuated. Boys showed a flatter trend at higher BMI levels, suggesting increased lean mass contribution. In contrast, girls exhibited a steeper and more consistent rise in %BF with increasing BMI.

These findings indicate that BMI alone may underestimate adiposity in girls and overestimate it in physically active boys, especially during mid- to late adolescence.

### Multivariate analysis: height, weight, BMI, and body fat percentage

**Figure 9:**
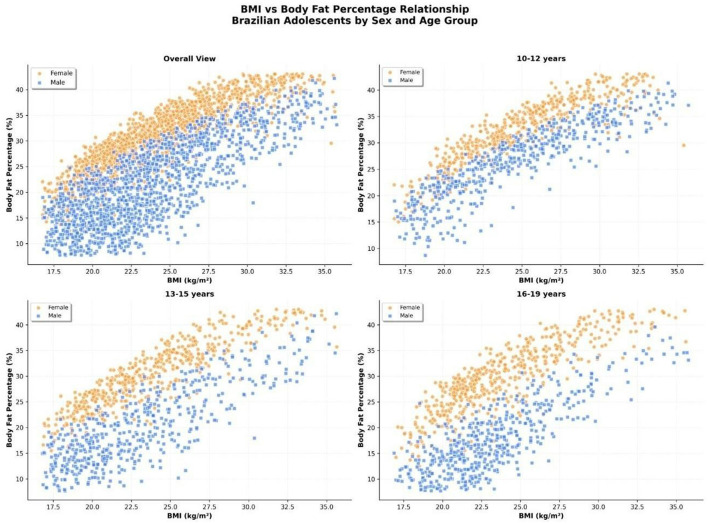
Multivariate scatterplot showing height (*x*-axis) vs. weight (*y*-axis) among Brazilian adolescents, stratified by sex (orange = female; blue = male) and age groups (10–12, 13–15, and 16–19 years). Dot size indicates body mass index (BMI), and dot color reflects body fat percentage (%BF), with a spectrum from purple/blue (lower %BF) to yellow/green (higher %BF). BMI and weight increased with height across all groups, but %BF distribution varied notably by sex and age.

A multivariate scatterplot was used to visualize the distribution of weight (*y*-axis) vs. height (*x*-axis), stratified by sex and age group, with body mass index (BMI) represented by the size of the dots and body fat percentage (%BF) by the color gradient (from purple/blue = lower %BF to yellow/green = higher %BF; Figure 9).

In the overall view, BMI values (marker size) increased proportionally with weight and height, but %BF (color) showed greater variability, particularly in individuals with similar BMI. This highlights the heterogeneity of body composition among adolescents, emphasizing that BMI does not fully capture adiposity.

When disaggregated by age group:

- 10–12 years: boys and girls exhibited overlapping ranges of height and weight, but girls had consistently higher %BF, even at lower BMI ranges.
- 13–15 years: height and weight dispersion increased, with boys tending to show larger body sizes (higher height and weight), but still lower %BF than girls. BMI varied widely, especially among girls.
- 16–19 years: the sexual dimorphism became accentuated. Boys appeared taller and heavier, with larger BMI values (larger dots), but lower fat percentages (cooler colors). Girls in the same BMI range displayed warmer colors, indicating greater fat accumulation.

These findings underscore the need to interpret BMI in conjunction with body composition indicators, especially during adolescence when growth and sexual maturation affect fat and lean mass distributions differently by sex.

### Detailed body composition analysis by sex and age group

**Figure 10:**
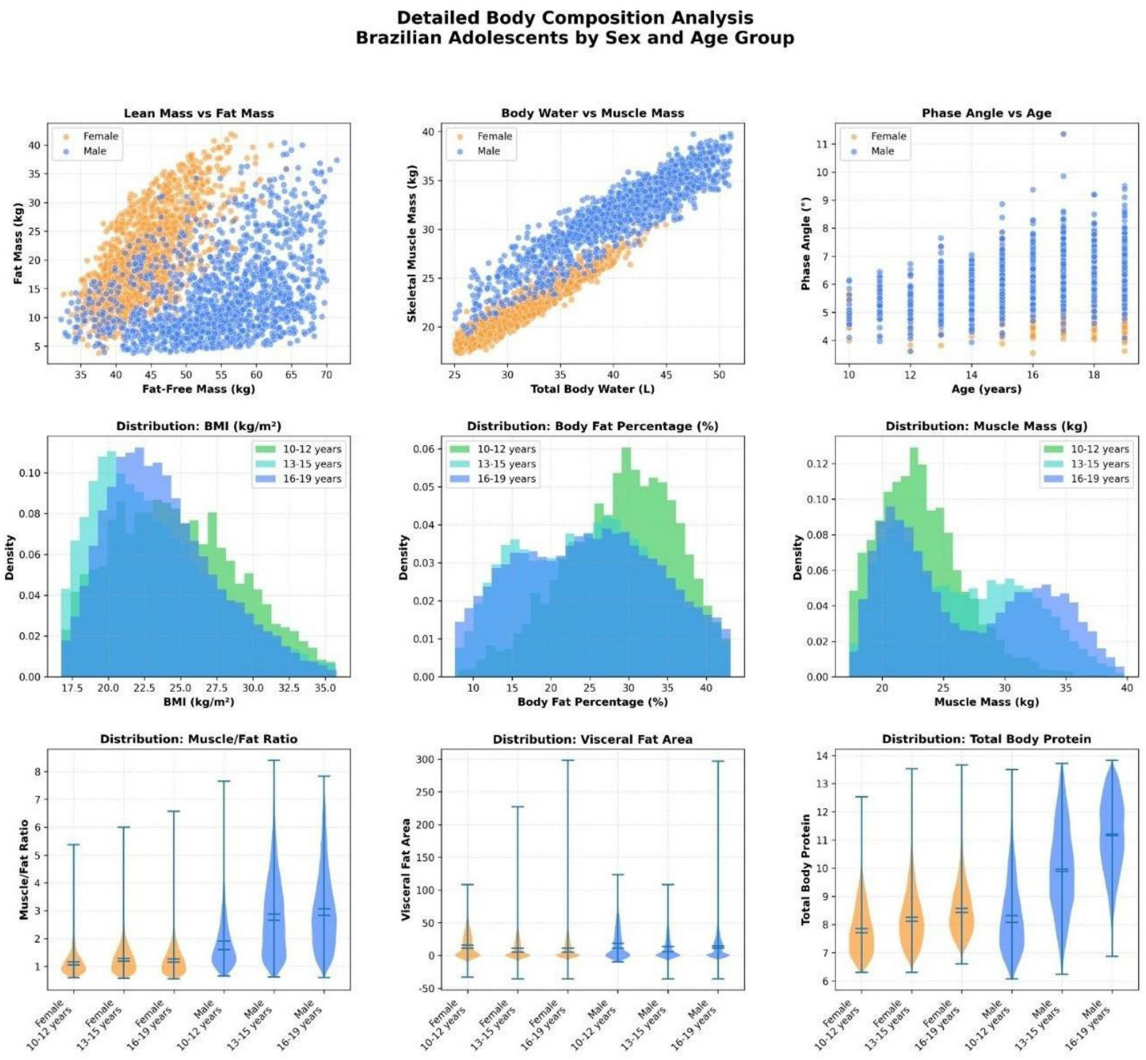
Comprehensive plots summarizing body composition among Brazilian adolescents (*N* = 59,000) by sex and age group. **(Top row)** scatterplots of fat-free mass vs. fat mass, total body water vs. skeletal muscle mass, and phase angle vs. age. **(Middle row)** histograms showing distributions of body mass index (BMI), body fat percentage (%BF), and skeletal muscle mass across three age groups (10–12, 13–15, and 16–19 years). (Bottom row) violin plots of muscle-to-fat ratio, visceral fat area, and total body protein, stratified by sex and age group. Males consistently showed higher muscle mass, water, protein, and phase angle values, while females exhibited higher fat percentages and lower muscle-to-fat ratios.

Figure 10 provides a comprehensive overview of body composition among Brazilian adolescents, with nine subplots detailing the interrelationships between key variables.

- Fat-free mass vs. fat mass: males exhibited significantly higher fat-free mass for any given fat mass, indicating greater lean tissue development, while females showed higher fat accumulation at similar lean mass levels.
- Total body water vs. skeletal muscle mass: a strong linear association was observed, especially among males. The slope appeared steeper in boys, reinforcing the notion that muscle mass contributes substantially to total body water content.
- Phase angle vs. age: phase angle, a marker of cellular integrity and health, increased progressively with age, particularly in boys, suggesting maturational gains in cell function and membrane integrity during adolescence.
- IMC, %BF, and muscle mass distributions (by age group): histograms revealed a rightward shift in BMI and muscle mass with age, especially among males. In contrast, the %BF distribution was more dispersed in girls across all age groups.
- Muscle-to-fat ratio: violin plots demonstrated a significantly higher muscle-to-fat ratio in males across all age groups, while females showed narrower distributions with lower median values.
- Visceral fat area: males had higher visceral fat values across all age groups. However, the absolute levels remained low, consistent with the adolescent population.
- Total body protein: protein mass followed the same pattern of muscularity, with higher values and greater dispersion in males, reflecting their enhanced lean mass.

Together, these findings highlight the sexual dimorphism and maturational progression of body composition components during adolescence, supporting the use of detailed segmental body composition metrics beyond BMI alone.

### Radar profiles of body composition by sex and age group

**Figure 11:**
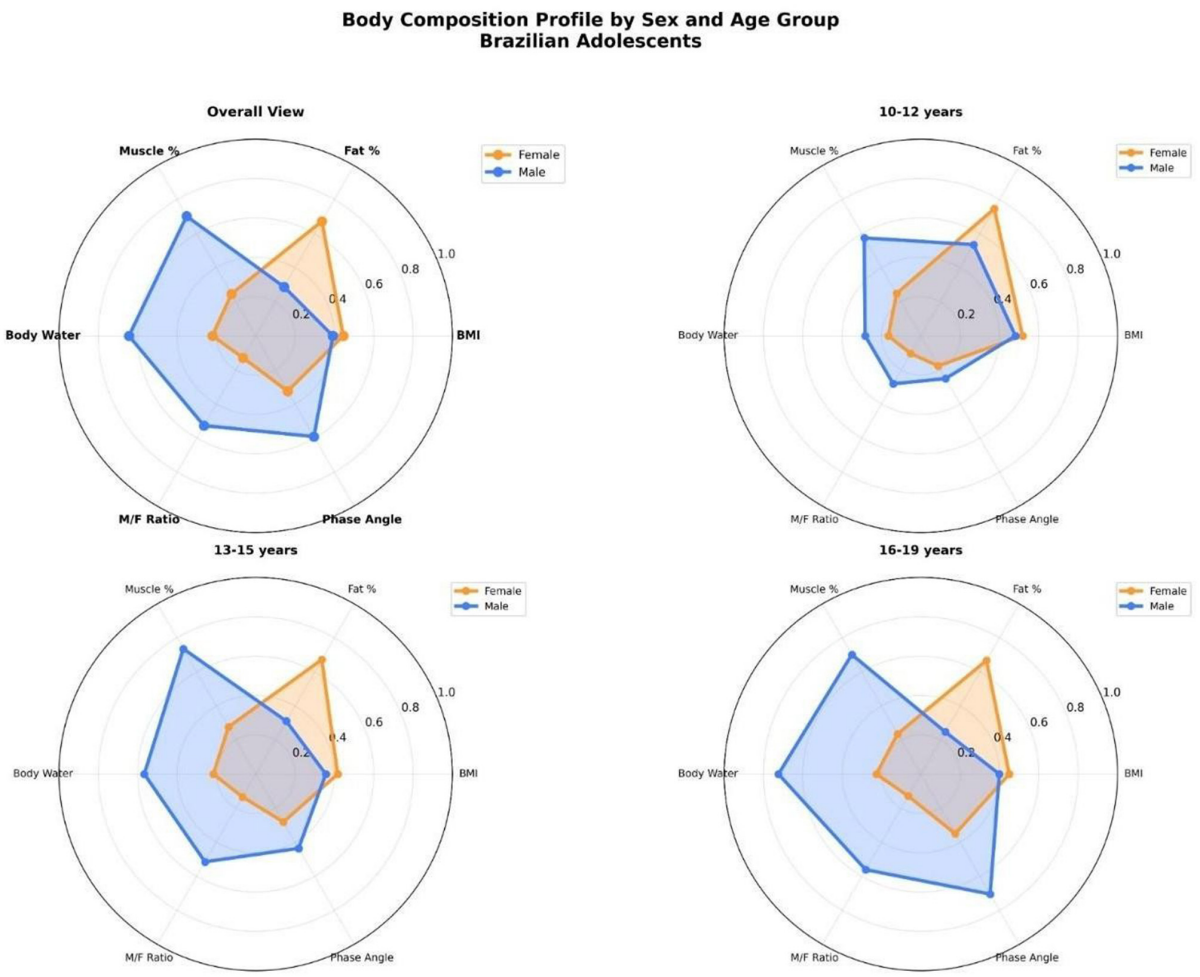
Normalized radar plots comparing body composition profiles between female (orange) and male (blue) adolescents across three age groups (10–12, 13–15, and 16–19 years), plus an overall view. Variables include BMI, body fat percentage, muscle percentage, total body water, muscle-to-fat ratio (M/F), and phase angle. Each axis is normalized from 0 to 1. The plots highlight distinct sex- based physiological trajectories during adolescence.

Figure 11 illustrates radar plots comparing normalized body composition parameters between male and female Brazilian adolescents across three age groups. The variables include: BMI, % body fat, % muscle mass, total body water, muscle-to-fat ratio (M/F), and phase angle.

- Sexual dimorphism: In all age groups, boys exhibited higher values in muscle mass, total body water, muscle-to-fat ratio, and phase angle. In contrast, girls consistently showed higher body fat percentages.
- Age progression: with increasing age, boys demonstrated progressive increases in muscularity-related variables, while girls showed relatively stable or slightly increasing fat mass values. The divergence in body composition profiles became more pronounced in the 16–19 years group.
- Phase angle and hydration: notably, boys showed greater values for phase angle and total body water from early adolescence onward, suggesting more favorable cellular health and hydration status.
- BMI similarity: BMI values remained relatively close between sexes within each age group, highlighting the limitation of BMI as a standalone metric for assessing sex-specific differences in body composition.

These radar plots emphasize the multidimensional nature of pubertal body composition changes and support the use of multi-variable assessments over unidimensional metrics like BMI.

Table 2 reports sex- and age-specific percentiles (P5, P25, P50, P75, P95) for key anthropometric and body composition variables, providing the full normative reference values.

**Table 2:** Full percentile values by sex and age for key variables.

**Age group**	**Sex**	***n***	**Mean ±SD**	**P5**	**P25**	**P50**	**P75**	**P95**
10–12 years	Male	1,885	153.7 ± 8.0	140.0	148.0	153.0	159.0	170.0
10–12 years	Female	1,544	154.8 ± 6.7	142.0	150.0	155.0	159.0	168.0
13–15 years	Male	6,729	168.6 ± 8.3	152.0	163.0	169.0	174.0	183.0
13–15 years	Female	6,375	161.6 ± 6.1	150.1	157.0	161.0	165.0	173.5
16–19 years	Male	18,753	174.6 ± 6.3	162.3	170.0	175.0	179.0	186.0
16–19 years	Female	14,953	163.1 ± 6.2	152.0	159.0	163.0	167.0	175.0
**Weight (kg)**								
10–12 years	Male	1,885	57.1 ± 14.0	37.9	46.1	54.4	65.8	88.2
10–12 years	Female	1,544	59.1 ± 12.1	41.0	49.6	57.6	66.4	85.1
13–15 years	Male	6,729	65.4 ± 15.3	42.9	54.0	62.8	74.4	100.0
13–15 years	Female	6,375	62.0 ± 13.1	43.5	52.0	59.9	69.8	90.8
16–19 years	Male	18,753	69.2 ± 14.8	47.0	58.0	67.0	78.0	102.0
16–19 years	Female	14,953	62.8 ± 12.9	44.0	53.0	61.0	71.0	88.0
**BMI (kg/m** ^2^ **)**								
10–12 years	Male	1,885	24.0 ± 5.0	16.8	20.0	23.2	27.2	34.8
10–12 years	Female	1,544	24.6 ± 4.4	18.0	21.2	24.0	27.2	33.2
13–15 years	Male	6,729	23.0 ± 4.7	16.2	19.5	22.2	25.8	32.8
13–15 years	Female	6,375	23.7 ± 4.2	17.5	20.5	23.0	26.2	32.0
16–19 years	Male	18,753	22.6 ± 4.1	16.8	19.5	21.8	24.8	31.0
16–19 years	Female	14,953	23.6 ± 4.0	17.8	20.5	23.0	26.0	31.5
**Body fat (%)**								
10–12 years	Male	1,885	18.2 ± 8.9	6.0	11.5	17.0	23.8	36.0
10–12 years	Female	1,544	25.8 ± 7.8	13.0	20.0	25.5	31.0	40.5
13–15 years	Male	6,729	16.1 ± 8.2	4.5	9.5	14.8	21.5	33.0
13–15 years	Female	6,375	24.2 ± 7.5	12.0	18.5	24.0	29.5	38.0
16–19 years	Male	18,753	13.8 ± 7.0	4.0	8.5	12.5	18.0	28.0
16–19 years	Female	14,953	21.8 ± 7.2	10.5	16.5	21.5	26.8	35.0

## Discussion

This nationwide analysis of 59,000 multi-frequency bioimpedance assessments provides one of the most comprehensive portraits of adolescent body composition reported in Latin America. By jointly describing body fat percentage (%BF), fat-free and skeletal muscle mass, total body water, phase angle (PhA), and the muscle-to-fat ratio (MFR), we delineate clear age- and sex- specific trajectories that mirror physiological maturation and emerging cardiometabolic risk. Collectively, these findings reinforce the inadequacy of body mass index (BMI) alone for adolescent surveillance and support the routine use of composition-based indices.

### Sex-specific trajectories and physiological plausibility

Consistent with pubertal endocrinology, boys showed progressive gains in lean/muscle compartments and PhA, whereas girls maintained higher adiposity and lower MFR, especially from mid- to late adolescence (2, 13–15). The monotonic rise (boys) and attenuation/plateau patterns (girls) we observed align with expected anabolic effects of testosterone and the lipogenic influence of estrogens, supporting the physiological plausibility of the curves. The scale and diversity of the cohort strengthen the robustness and external relevance of these patterns, complementing smaller, region-specific studies from Brazil and other Latin American settings and bringing them closer to reference initiatives reported in high-income countries (8, 16, 17).

### Clinical interpretation beyond BMI

BMI conflates fat and lean mass and can obscure opposite risk trajectories during puberty (18, 19). In our data, adolescents with comparable BMI displayed wide dispersion in PhA and MFR, underscoring the added value of composition- based markers for risk stratification. Early deviations—low muscle quantity/quality and higher adiposity—track with insulin resistance and clustered cardiometabolic risk in youth (20–22); higher lean-to-fat ratios are associated with more favorable lipid, inflammatory, and glycemic profiles (23). Incorporating PhA (a proxy of cellular integrity and hydration) and MFR into routine assessments adds actionable granularity; both have been associated with fitness, nutritional status, and adverse outcomes in pediatric contexts (24–26), and low MFR may signal a “sarcopenic obesity” phenotype even at normal BMI (27, 28). In this light, we prioritize PhA, SMMI, and MFR as primary reference outputs, with %BF presented as secondary and interpretation-cautioned.

### Population and policy implications

Brazil faces the dual burden of undernutrition and excess weight in adolescence amid declining physical activity and rising consumption of ultra- processed foods (29). Age- and sex-stratified reference curves for PhA, SMMI, and MFR can sharpen screening, enable tailored counseling, and improve program monitoring across school, primary-care, and community settings. Because these indices are derived from raw measurements or transparent transformations, they are less sensitive to device-specific assumptions and are therefore pragmatic first-line tools for surveillance and clinical follow-up.

### Provenance, governance, and transparency of the analytic cohort

In response to reviewer concerns about reproducibility and selection bias, we explicitly document the data-governance pathway. The operational stream of routine BIA assessments undergoes the vendor's standardized privacy and quality-assurance pipeline—compliant with Brazil's LGPD—to generate a frozen, de-identified research extract. Authors did not recruit participants, did not have access to the master pool or operational pre-cleaning logs, and could not curate records at the individual level. To maximize transparency within these constraints, we prespecified plausibility and deduplication rules (anthropometry and impedance thresholds; robust BIVA on R/H and Xc/H; deterministic duplicate checks) and re-applied them to the frozen extract, which yielded zero additional exclusions because all records already satisfied the validity criteria. Figure 1 summarizes this governance/analytic flow (rather than a traditional attrition diagram), and Table 1 reports the macro-regional and recruitment-context distributions of the analytic cohort.

### Representativeness and generalizability across Brazil

The dataset spans all five Brazilian macro-regions and multiple recruitment contexts (schools, clinics/primary care, private practices, gyms/fitness centers, community programs), but participation was not proportional to national census distributions. Relative participation was highest in the Southeast, followed by the Center-West and South, with smaller contributions from the Northeast and North. Such asymmetry can shift absolute percentile levels for specific strata and must be considered when applying the curves to local settings. We therefore (i) report macro-regional/context breakdowns in Table 1, (ii) recommend local calibration and/or regional external validation when feasible, and (iii) frame our curves as nationwide device-based references that reflect the full stream of eligible adolescent assessments captured by the network during the study window.

### Device dependence of %BF and implications for validation

A central methodological limitation—also highlighted by the reviewer—is the device dependence of %BF. The underlying regression coefficients are the intellectual property of the manufacturer and cannot be disclosed under the confidentiality agreement; this is standard across several commercial analyzers. To preserve interpretability without breaching confidentiality, we (i) treat %BF as a secondary output, (ii) elevate non-proprietary indices (PhA, SMMI, MFR) as primary references, and (iii) add an explicit interpretation note in the Results indicating that %BF percentiles should be used with caution until externally validated against a criterion method (e.g., DXA) in a representative Brazilian adolescent subsample.

### Strengths of the present analysis

Beyond scale and coverage, our approach combines standardized acquisition and device quality controls across participating centers; an explicit, prespecified QC pipeline re-applied to the frozen extract; modeling with GAMLSS (LMS) to derive smooth, age- and sex-specific percentiles and to capture non- linear maturational patterns; and integration of functional/structural markers (PhA, MFR) that add clinical granularity over and above BMI. Together, these elements help mitigate common sources of bias in large observational datasets and support the internal validity and practical utility of the reference curves.

### Limitations and sensitivity to unmeasured factors

Important caveats warrant emphasis. First, the absence of direct pubertal staging (e.g., Tanner) limits the precision with which biological maturation can be separated from chronological age. Second, the cross-sectional design precludes inference on intraindividual change; longitudinal follow-up is needed to link percentile trajectories with clinical outcomes (e.g., incident hypertension, dysglycemia, or diminished cardiorespiratory fitness). Third, behavioral and sociodemographic covariates (dietary patterns, physical activity, socioeconomic status, ethnicity/skin color) were not available in the research extract and likely modulate body composition during adolescence. Furthermore, regional distribution was uneven, with relative under-representation of the Northeast and over-representation of the Central-West, which constrains unrestricted generalization of these percentiles to the entire Brazilian territory. Fourth, as noted, the governance architecture that protects privacy and prevents selective sampling also prevents reporting stage-wise upstream exclusion tallies or the characteristics of excluded records. Finally, device dependence of %BF constrains cross-device comparability until external validation is completed.

### Pragmatic guidance for clinical and programmatic use

In light of these strengths and limitations, we offer pragmatic guidance. For screening and surveillance, PhA, SMMI, and MFR should be prioritized because they are either directly measured or derived via transparent transformations and thus more portable across services. When %BF is considered, clinicians and program managers should interpret percentiles cautiously, avoid cross-device comparisons, and prefer within-device longitudinal assessment until independent validation data become available. For borderline cases (e.g., normal BMI with low MFR or low PhA), early, low-risk interventions—physical-activity counseling emphasizing resistance training, nutrition guidance focused on protein adequacy and overall diet quality, and sleep hygiene—are reasonable while awaiting confirmatory evaluation.

The revision incorporates the reviewer's requests to (i) disclose macro- regional and recruitment-context distributions and discuss their impact on generalizability; (ii) replace the attrition-style figure with a governance/analytic- flow schematic that clarifies why upstream exclusion counts are unavailable and documents zero additional exclusions after re-applying prespecified QC rules; (iii) acknowledge the proprietary nature of the %BF algorithm, elevate non- proprietary indices as primary, and state the need for external validation prior to routine use; and (iv) document independence of analysis given the declared conflict of interest (expanded COI statement in the manuscript).

### Future directions

Two near-term priorities follow directly from the remaining gaps: (1) governance-compliant external validation of the device-dependent %BF against a criterion method (e.g., DXA) in a representative Brazilian adolescent subsample, with pre-registration of protocol and a Bland–Altman framework to quantify bias, precision, and total error; and (2) regional calibration/validation to quantify geographic heterogeneity and test portability of cut-points. In the medium term, longitudinal cohorts should link percentile positions and transitions (e.g., crossing centiles) with clinical outcomes and fitness trajectories, refine actionable thresholds for PhA, SMMI, and MFR, and evaluate whether combining these indices with simple functional tests (e.g., grip strength, shuttle run) improves prediction. Privacy-preserving aggregation strategies could enable incorporation of macro-regional tags and behavioral covariates in future releases without compromising LGPD compliance.

## Conclusion

We provide national reference percentiles and smoothed curves for PhA, SMMI, and MFR—immediately useful for clinical and public-health applications and less sensitive to device-specific assumptions—and offer %BF curves as secondary, with explicit caution. The clinical applicability of the percentile curves—particularly for %BF—depends on independent validation of the predictive equation and confirmation of sample representativeness. Only after these conditions are addressed should the curves be considered for routine use in nutritional surveillance and clinical practice in Brazil.

## Data Availability

The data analyzed in this study was obtained from Tera Science Inc. The original dataset cannot be commercially explored. Requests to access these datasets should be directed to fernando@terascience.com.br.
