# Correction: Establishing percentiles for phase angle, skeletal muscle mass index, and muscle-to-fat ratio in Brazilian adolescents: a bioelectrical impedance analysis study with 59,000 participants

**DOI:** 10.3389/fnut.2026.1863696

**Published:** 2026-05-05

**Authors:** Frontiers Production Office

**Affiliations:** Frontiers Media SA, Lausanne, Switzerland

**Keywords:** adolescents, body composition, BIA, reference values, phase angle, bioelectrical impedance analysis

The title of this article was erroneously given as: “Establishing national normative values for adolescent body composition in Brazil using bioelectrical impedance analysis (BIA): a cross-sectional study of 59,000 adolescents”. The correct title of the article is “Establishing percentiles for phase angle, skeletal muscle mass index, and muscle-to-fat ratio in Brazilian adolescents: a bioelectrical impedance analysis study with 59,000 participants.”

The original version of this article has been updated.

